# Intraduodenal Administration of Intact Pea Protein Effectively Reduces Food Intake in Both Lean and Obese Male Subjects

**DOI:** 10.1371/journal.pone.0024878

**Published:** 2011-09-13

**Authors:** Maartje C. P. Geraedts, Freddy J. Troost, Marjet J. M. Munsters, Jos H. C. H. Stegen, Rogier J. de Ridder, Jose M. Conchillo, Joanna W. Kruimel, Ad A. M. Masclee, Wim H. M. Saris

**Affiliations:** 1 Department of Human Biology, Maastricht University Medical Center, Maastricht, The Netherlands; 2 Department of Internal Medicine, Division of Gastroenterology and Hepatology, Maastricht University Medical Center, Maastricht, The Netherlands; 3 Nutrition and Toxicology Research Institute Maastricht, Maastricht, The Netherlands; Clermont Université, France

## Abstract

**Background:**

Human duodenal mucosa secretes increased levels of satiety signals upon exposure to intact protein. However, after oral protein ingestion, gastric digestion leaves little intact proteins to enter the duodenum. This study investigated whether bypassing the stomach, through intraduodenal administration, affects hormone release and food-intake to a larger extent than orally administered protein in both lean and obese subjects.

**Methods:**

Ten lean (BMI:23.0±0.7 kg/m^2^) and ten obese (BMI:33.4±1.4 kg/m^2^) healthy male subjects were included. All subjects randomly received either pea protein solutions (250 mg/kg bodyweight in 0.4 ml/kg bodyweight of water) or placebo (0.4 ml/kg bodyweight of water), either orally or intraduodenally via a naso-duodenal tube. Appetite-profile, plasma GLP-1, CCK, and PYY concentrations were determined over a 2 h period. After 2 h, subjects received an *ad-libitum* meal and food-intake was recorded.

**Results:**

CCK levels were increased at 10(*p<0.02*) and 20(*p<0.01*) minutes after intraduodenal protein administration (IPA), in obese subjects, compared to lean subjects, but also compared to oral protein administration (OPA)(*p<0.04*). GLP-1 levels increased after IPA in obese subjects after 90(p<0.02) to 120(*p<0.01*) minutes, compared to OPA. Food-intake was reduced after IPA both in lean and obese subjects (-168.9±40 kcal (*p<0.01*) and −298.2±44 kcal (*p<0.01*), respectively), compared to placebo. Also, in obese subjects, food-intake was decreased after IPA (−132.6±42 kcal; *p<0.01*), compared to OPA.

**Conclusions:**

Prevention of gastric proteolysis through bypassing the stomach effectively reduces food intake, and seems to affect obese subjects to a greater extent than lean subjects. Enteric coating of intact protein supplements may provide an effective dietary strategy in the prevention/treatment of obesity.

## Introduction

Food ingestion triggers a number of stimuli, such as the release of the gastrointestinal hormones cholecystokinin (CCK), glucagon-like peptide 1 (GLP-1) and peptide YY (PYY). These hormones are known to be involved in the modulation of appetite sensations. CCK is produced by I-cells in the duodenal and jejunal mucosa, and is secreted in response to luminal food compounds, especially lipids and proteins [1]. GLP-1 and PYY are produced primarily by the L-cells in the more distal small intestine and colon. Ingested nutrients stimulate CCK-, GLP-1-, and PYY secretion indirectly by neurohumoral mechanisms, e.g. feedback mechanisms of hormones from a more distal part of the small intestine, as well as by direct sensing mechanisms at the intestinal mucosa [2], [3].

Previously, it was demonstrated that the plasma levels of GLP-1 were elevated in obese rats, compared to lean rats [4]. Others found that levels of ghrelin and obestatin were decreased in obese children, compared to lean children [5]. Also, PYY levels are lower in obese subjects compared to lean subjects [6]. These data indicate that there are significant differences between lean and obese subjects with respect to hormone release, and that the gut may respond different to ingested nutrients in obese subjects, compared to lean subjects.

Among all properties of food, the total energy content and the macronutrient composition appears to be one of the major determinants of the control of food intake. Recent literature points to the effect of dietary protein in reducing food intake by improving satiety sensations [7], [8]. It seems that proteins have the highest satiating effect when compared to carbohydrates and in particular fats in humans and rats [9], [10], although the nature of the protein can influence its satiating effects. In most cases, high-protein meals increase feelings of satiety and decrease subsequent energy intake compared with high-carbohydrate or high-fat meals [11].

From previous work it is well known that proteins have the ability to affect food intake and appetite in several species, including humans [7], [12], [13], [14]. A variety of physiological mechanisms activated by protein ingestion may act in concert to exert their satiating effect. One of the physiological processes through which proteins appear to induce satiety is by stimulating satiety hormone secretion [15]. However, little is known about the satiating properties of proteins originating from different sources.

In previous studies we showed that proteins and especially codfish, egg, pea, and wheat exert strong effects on satiety hormone release in an *in vitro* setting. Remarkably, the hydrolysates showed weaker effects with respect to hormone release, than the intact protein [16]. We also demonstrated that these intact proteins exert different effects on satiety hormone release in human duodenal tissue. Exposing intestinal tissue to intact pea and wheat protein induced an increase in the release of both CCK and GLP-1, whereas al the other tested proteins did not affect the hormone release [17].

It was shown previously in rats that intraduodenal infusion of dietary protein resulted in increased plasma levels of CCK compared to intragastric infusion [18]. We hypothesize that 1) intraduodenal infusion of intact dietary proteins in humans will induce stronger effects on satiety hormone release and satiation, when compared to orally ingested dietary proteins, and that 2) there are differences in hormone secretion between lean and obese subjects. To investigate this, the effects of different administration routes of pea protein on plasma satiety hormone levels, feelings of hunger and satiety, and food intake were investigated in both lean and obese subjects.

## Results

### Satiety and well-being scores

**Figure 1:**
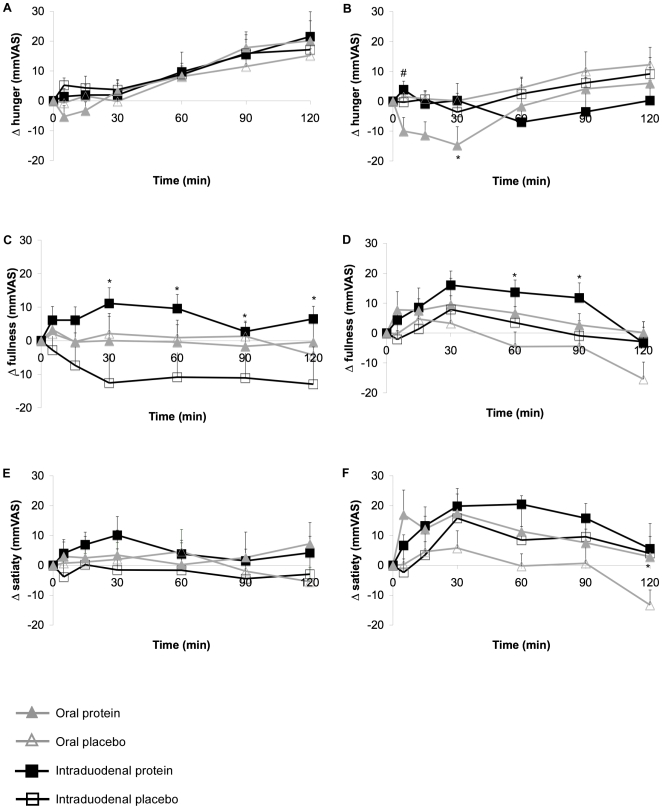
Changes in hunger, fullness, and satiety (mmVAS) after oral and intraduodenal treatments with pea protein and placebo. Feelings of hunger did not change in lean subjects after between treatments (A), whereas in obese subjects, hunger decreased at 30 minutes after the oral pea protein ingestion (B). Feelings of fullness were increased after intraduodenal pea protein treatment for both lean (C) and obese (D) subjects. Satiety did not change over time when compared to placebo treatments for both lean (E) and obese (F) subjects. Values are expressed as mean ± SEM. * Significantly different from placebo treatment (p<0.05). # Significantly different from oral protein treatment (p<0.05).

All fasting scores did not differ between treatments for both lean and obese subjects. There was also no difference between placebo treatments in both groups. For lean subjects, feelings of hunger increased over time (**figure 1A**), but there were no significant differences between treatments. Obese subjects reported decreased feeling of hunger after 30 minutes with the oral protein treatment (figure 1B), compared to the other treatments. After 120 minutes, no differences were observed in feelings of hunger between treatments in obese subjects. Also, obese subjects reported to feel less hungry at 120 minutes after intraduodenal protein treatment when compared to lean subjects with the same treatment.

Even though we did not see differences in feeling of hunger in lean subjects, these subjects felt more full after the intraduodenal treatment (figure 1C), compared to the placebo treatment. There were no differences in the oral treatment. Between 60 and 90 minutes, obese subjects felt more full after intraduodenal protein treatment (figure 1D), compared to placebo treatment. However, there were no differences between the lean and obese group. Satiety feelings did not change over time for lean subjects (figure 1E), whereas for obese subjects, satiety feelings were significantly increased at 120 minutes after oral protein ingestion, compared to oral placebo ingestion (figure 1F).

There were no differences in feelings of thirst, desire to eat, or feelings of well-being between the treatments and between the groups. Subjects scored no feelings of nausea, intestinal cramps, diarrhea, headache, or dizziness during any treatment.

### Plasma Ghrelin

Baseline levels of total Ghrelin did not differ between study days, and between groups (data not shown). Placebo treatments or protein treatments did not affect the total Ghrelin levels in both groups (data not shown).

### Plasma CCK

**Figure 2:**
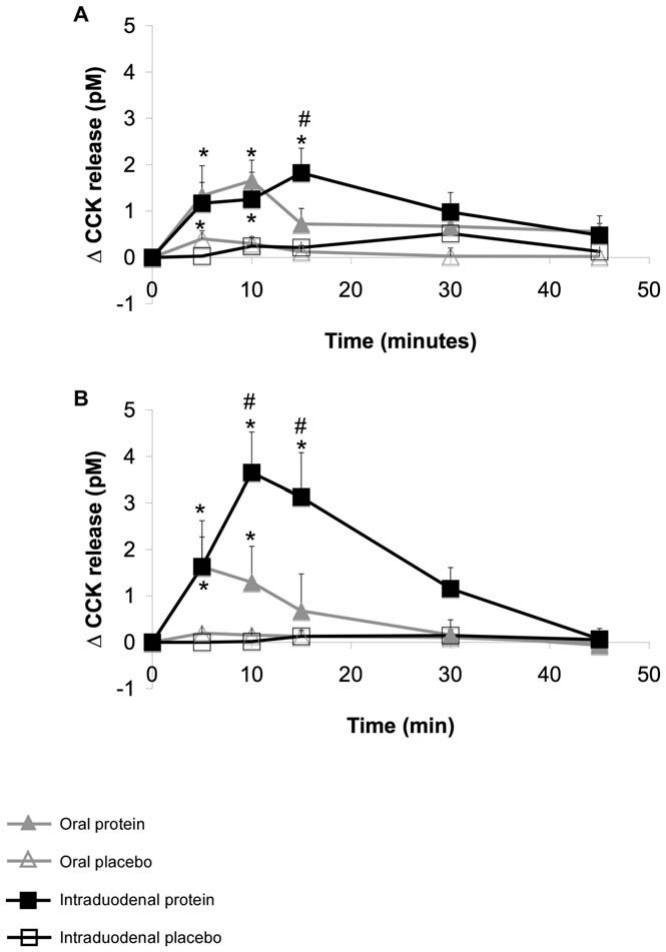
Changes in plasma CCK release (pM) after oral and intraduodenal treatments with pea protein and placebo. CCK release was increased during the first 10 minutes after oral pea protein ingestion in both lean (A) and obese (B) subjects. However, after intraduodenal pea protein administration, CCK levels remained elevated for 15 minutes, and the levels were higher when compared to oral protein administration. Values are expressed as mean ± SEM. * Significantly different from placebo treatment (p<0.05). # Significantly different from oral protein treatment (p<0.05).

Baseline plasma CCK concentrations did not differ between study days, and between groups (data not shown). Placebo treatments did not affect the plasma CCK levels in all treatments in both groups. Oral ingestion of pea protein in lean subjects (**figure 2A**) resulted in significantly increased levels of CCK for the first 10 minutes, whereas after intraduodenal infusion of pea, CCK levels where significantly increased during the first 15 minutes. After 30 minutes, CCK levels returned to baseline for both protein treatments. More pronounced differences were observed in obese subjects (figure 2B). After oral protein ingestion, CCK levels were significantly increased for the first 10 minutes, when compared to the placebo treatment. However, after intraduodenal administration of pea protein, CCK levels were significantly increased during the first 15 minutes, when compared to the placebo treatment, but also when compared to the oral treatments. Intraduodenal protein treatment showed stronger effects on CCK release in obese subjects than in lean subjects.

### Plasma active GLP-1

**Figure 3:**
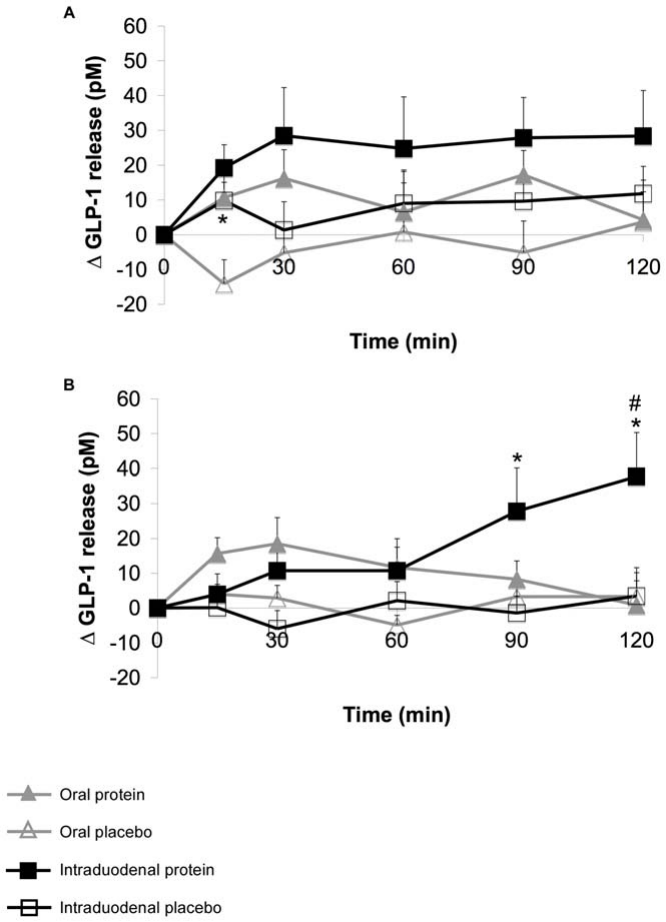
Changes in plasma GLP-1 release (pM) after oral and intraduodenal treatments with pea protein and placebo. GLP-1 levels did not change over time after pea protein ingestion in lean subjects (A), when compared to the placebo treatments. In obese subjects, GLP-1 levels were significantly increased from 90 minutes after intraduodenal pea protein administration until the end of the test (B). Oral pea protein administration did not affect GLP-1 release. Values are expressed as mean ± SEM. * Significantly different from placebo treatment (p<0.05). # Significantly different from oral protein treatment (p<0.05).

Baseline plasma GLP-1 concentrations did not differ between study days, and between groups (data not shown). Placebo treatments did not affect the plasma GLP-1 levels in all treatments in both groups. Both protein treatments showed no effect on GLP-1 release in lean subjects (**figure 3A**), with the exception of the oral protein treatment at time point 15 minutes. Here GLP-1 levels were significantly increased when compared to the placebo treatment. In obese subjects, the oral protein treatment did not affect GLP-1 release when compared to the placebo treatment (figure 3B). Intraduodenal protein administration in obese subjects resulted in significantly increased levels after 90 minutes until the end of the test, compared to the placebo treatment, but also compared to the oral treatment.

### Plasma PYY

**Figure 4:**
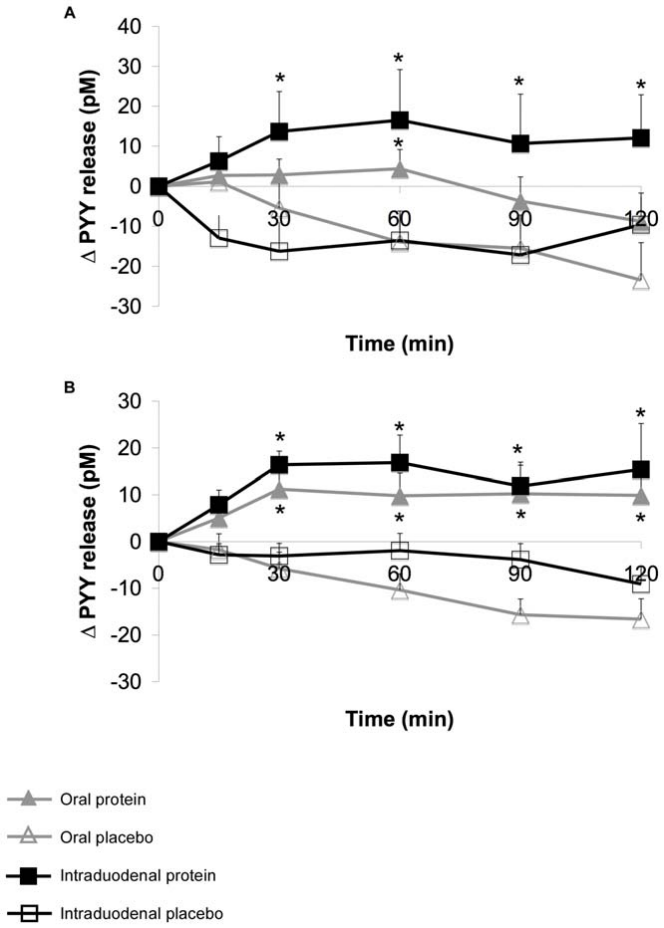
Changes in plasma PYY release (pM) after oral and intraduodenal treatments with pea protein and placebo. Oral pea protein ingestion did not affect PYY release in lean subjects (A), whereas intraduodenal pea protein administration significantly increased the release of PYY from 15 minutes after pea protein administration until the end of the test. In obese subjects (B), PYY release was significantly increased from 30 minutes until the end of the test for both protein treatments. Values are expressed as mean ± SEM. * Significantly different from placebo treatment (p<0.05). # Significantly different from oral protein treatment (p<0.05).

Baseline plasma PYY concentrations did not differ between study days, and between groups (data not shown). Placebo treatments did not affect the plasma PYY levels in all treatments in both groups. In lean subjects, intraduodenal protein administration induced stronger effects on PYY release compared to oral protein ingestion (**figure 4A**). PYY levels were increased after 15 minutes, until the end of the test, whereas during the oral treatment, PYY levels were only increased after 60 minutes, compared to the placebo. The effects of protein administration in obese subjects did not differ between oral and intraduodenal treatment (figure 4B). For both treatments, PYY levels were significantly increased after 30 minutes, until the end of the test, compared to the placebo treatment.

### Food intake

**Figure 5:**
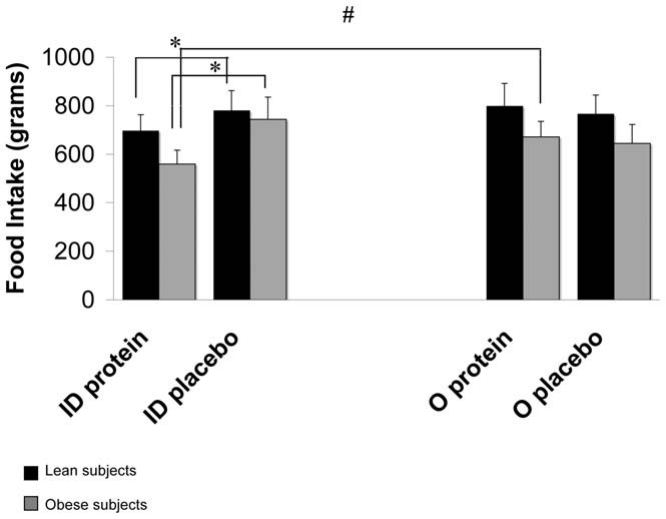
*Ad libitum* food intake (g) after oral and intraduodenal treatments with pea protein and placebo. Oral protein ingestion did not affect food intake. Both lean and obese subjects decreased food intake after intraduodenal protein administration compared to the placebo treatment. Moreover, obese subjects decreased their food intake also when compared to oral protein treatment, whereas this was not the case for lean subjects. ID: Intraduodenal treatment; O: oral treatment. Values are expressed as mean ± SEM****. * Significantly different from intraduodenal placebo treatment (p<0.05). # Significantly different from oral protein treatment (p<0.05).

Consumption of the *ad libitum* meal did not differ between lean and obese subjects. Orally ingested protein did not affect the consumption of the meal, whereas intraduodenally administered protein decreased food intake in both lean and obese subjects (-168.9 ± 40 kcal (*p<0.01*) and −298.2 ± 44 kcal (*p<0.01*), respectively). Obese subjects also consumed significantly less after the intraduodenal protein treatment when compared to the oral protein treatment (−132.6 ± 42 kcal; *p<0.01*) (**figure 5**).

## Discussion

This study demonstrated for the first time that in both lean and obese humans, intraduodenal administration of intact pea protein is effective in reducing food intake, whereas no differences between placebo and treatment were observed after oral administration of the protein. This effect was not seen when the protein was given orally to the same subjects. Also, intraduodenal infusion of protein resulted in stronger effects on CCK release in obese subjects than in lean subjects, whereas effects on GLP-1 and PYY release were comparable in both groups.

Digestion of proteins takes place in different stages. The initial phase of protein digestion and absorption occurs in the stomach, followed by protein digestion in the small intestine. Until now, it was commonly accepted that proteins that are more rapidly absorbed in the body (also called fast proteins) appear to have a larger effect on satiety than slow proteins, which coagulate in the stomach and induce lower levels of amino acids in the bloodstream [21]. In humans, it has been demonstrated that whey, a fast protein, was more satiating and induced stronger postprandial hormone release than casein, a slow protein [22]. It could also be possible that the ‘fast’ proteins are not digested well in the stomach, leaving more intact protein to enter the duodenum, which in turn might result in higher satiation. However, no literature is available on the levels of intact proteins that reach the duodenum.

In the present study, the effects of pea protein on food intake were studied. This protein contains large amounts of arginine, asparagine, and glutamine, and is digested by approximately 93% in the stomach [23]. The study of Diepvens et al showed that pea protein hydrolysates had a more pronounced effect on PYY release and feelings of hunger, compared to whey and whole milk proteins [12]. However, in a recent study from our group we demonstrated that intact pea proteins were more potent in stimulating hormone release from enteroendocrine cells, than protein hydrolysates and specific peptides (accepted in Molecular Food and Nutrition research, 2010). In another study, we demonstrated that intact pea and wheat protein stimulated CCK and GLP-1 release from human duodenal tissue to a greater extent than egg and codfish protein and negative control [24]. Unlike other common protein sources such as milk, soy, or wheat proteins, pea protein has a very low allergenic potential, which makes this protein more suitable for dietary interventions than wheat protein.

The present study shows that intact pea protein is more satiating than its digested products. However, we do not know whether the subjects compensated the decreased portion size with an increased portion size during the next meal. Also, the amount of protein given is relatively high. Subjects received 250 mg per kg of bodyweight, which is comparable with a fourth of the daily total protein intake (approximately 1 gram per kg of body weight per day [25]). It has previously been demonstrated that this amount of protein affects satiety [26], and therefore, this amount was also used in the present study. However, future studies should be designed to identify the lowest effective dose of this protein. Also, options for delivering intact proteins to the duodenum will have to be investigated. There are some studies describing coatings for enteric delivery, such as pH-triggered (micro)coatings, pressure-sensitive coatings, or time-release coatings [27], [28], [29], [30], [31]. For duodenal delivery of a large amount of protein, a pH-sensitive coating seems suitable. It should be noted that, under physiological circumstances, little intact protein will reach the duodenum. Administering large amounts of intact protein to the duodenum might result in decreased feelings of well-being. Here we demonstrated that these amounts of protein did not affect feelings of nausea, stomach ache, diarrhoea and other factors of well-being. As was demonstrated in the past, undigested lipids infused more distal in the ileum caused similar effects [2]. This phenomenon called ‘ileal brake’ could also be active in the present situation of undigested protein proximal in the small intestinal tract. The exact mechanisms are still not fully explained as in the lipid infusion studies as in our intact protein infusions not all gut hormones respond similar in relation to satiety response. Other local neurohumoral factors could be involved.

There are indications that obese subjects are less sensitive for satiety signals compared to lean subjects. In rats it was shown that the minimal effective dose of satiety hormones was 3–4 times greater in obese than in lean rats [32], [33]. Here we demonstrated that after the same protein load, CCK levels in obese subjects were higher when compared to lean subjects, whereas GLP-1 and PYY levels did not differ between both groups. This would indicate that there is an impaired balance between protein load sensitivity and release of CCK in obese subjects. Even though the CCK levels were higher, both lean and obese subjects decreased food intake following protein ingestion to the same extent. This suggests that obese subjects are less sensitive to the satiety signals. Although there are no indications that age might have an influence on the present results, we should take into consideration, that even though not significant, there is a trend that there is a age difference between lean and obese subjects used in the present study. We should also take into consideration that perhaps obese subjects are also more sensitive to external cues that influence the amount of food eaten [34]. Hence, the laboratory setting may have influenced eating behaviour in the obese subjects to a larger extent than the lean. However, there were no significant differences between lean and obese subjects in relation with the appetite ratings. VAS are often used to measure subjective appetite sensations and the validity and reproducibility has been shown in several studies [20], [35]. Both groups showed the same changes over time, which may also explain why both groups consumed the same amount of food.

In conclusion, food intake was decreased after intraduodenal protein administration in both lean and obese subjects, in contrast to food intake after oral ingestion of the protein. Although there were no differences in appetite ratings between both groups, we observed elevated levels of CCK in obese subjects, and GLP-1 and PYY were elevated in both groups. This suggests that by preventing gastric pea protein degradation may be an effective dietary strategy in the prevention and treatment of obesity. However, more studies will have to be performed to identify the lowest effective dose of the protein, and whether (micro)encapsulated proteins show the same effects on food intake. Also, long-term intervention studies will have to be performed to demonstrate the effects of intraduodenal pea protein administration on weight loss and weight maintenance.

## Methods

### Subjects

Twenty male subjects participated in this study. We included only male subjects, because with female subjects, the hormonal fluctuations may influence the satiety hormone levels during this study. The subjects were recruited by advertisements on boards at the university and in local newspapers. Selection took place according to health criteria (no diabetes, no gastrointestinal diseases, and no medical treatment) and body weight (BW) criteria (for lean subjects: body mass index (BMI) 18–25 kg/m^2^, and for obese subjects: BMI>30 kg/m^2^). Baseline characteristics of the subjects are presented in **table 1**. The nature and risks of the experimental procedure were explained to the subjects, and all subjects gave their written informed consent. This study was conducted according to the guidelines laid down in the Declaration of Helsinki [19]. The Medical Ethical Committee of the University Hospital Maastricht approved all procedures involving human subjects.

**Table 1:** Subject characteristics at baseline.

	Lean (n = 10)	Obese (n = 10)
Age (years)	25±2	41±6 #
BMI (kg/m^2^)	23.0±0.7	33.4±1.4*
HbA1c (%)	5.0±0.1	5.0±0.1
Basal glucose (mmol/L)	4.8±0.2	4.5±0.1

All data are mean ± SEM.

• Difference between lean and obese subjects (p<0.05).

# p-Value = 0.054 between lean and obese subject.

### Nasointestinal catheter

Participants were intubated with a 145 cm nasoduodenal catheter (Flocare Bengmark, Nutricia, Zoetermeer, The Netherlands). This catheter is designed for transnasal feeding directly into the duodenum or jejunum. The tip of the catheter was lubricated with lidocaïne gel to sedate the nostril. The catheter was introduced into the stomach and the tip was positioned in the duodenum under radiological guidance and verification.

### Experimental design

In this single-blind, randomized controlled crossover design, each participant participated in four experiments on four occasions with 1 week between visits. All subjects received a standardized evening meal (9 g protein, 39,5 g carbohydrates, 16 g fat per meal) on the day prior to the test day to standardize macronutrient intake. On each occasion, subjects arrived after fasting overnight. The nasoduodenal catheter and an intravenous blood sampling catheter were placed during each test day, and a fasting blood sample was taken. Subjects then completed an appetite and well-being questionnaire. The test-drink (for composition see ‘dietary protocol’) was consumed at time 0. During oral consumption of the test drink, subjects were instructed to consume the drink within 5 minutes. During intraduodenal infusion of the drink, the fluid was infused for 30 minutes, at a rate depending on the volume of the test-drink, which was based on total body weight of the volunteer. Subsequent blood samples were collected at 0, 5, 10, 15, 30, 45, 60, 75, 90, and 120 min, respectively. An appetite questionnaire was completed 0, 5, 15, 30, 60, 90, and 120 min, respectively. A well-being questionnaire was completed 0, 5, 30, 60, 90, and 120 min, respectively. After 120 min, the nasoduodenal catheter and the intravenous catheter were removed, and subjects were offered an *ad libitum* meal approximately 10 minutes after the removal of the catheter. Instructions were given to eat until comfortably satiated.

### Dietary protocol

The protein tested was provided as part of a drink (4 ml per kg of bodyweight), which contained either pea protein (250 mg per kg of body weight)(Dutch Protein Services, Tiel, The Netherlands) or only water as placebo. This concentration of protein was chosen, because it is comparable with the amount of protein that one meal should contain on average. The drinks were uniformly flavored with 5 g cream vanilla flavor (Quest International, Naarden, The Netherlands) per liter beverage to mask the taste of the pea protein. The drinks were provided in covered flasks so that the subjects did not know which product they received.

The *ad libitum* meal consisted of Lasagne Bolognese (671 kJ, 7.5 g protein, 12.3 g carbohydrates, 9.0 g fat per 100 grams). Food was weighed prior to the meal and after the subjects left the laboratory. Food intake was assessed by difference.

### Questionnaires

The appetite questionnaire was a Visual Analogue Scale questionnaire (VAS, on a 100 mm scale) with questions about feelings of hunger, fullness, satiety, thirst, and desire to eat [20]. Opposing extremes of each feeling were described at either end of a 100-mm horizontal line, and subjects marked the line to indicate how they felt at that moment.

Well-being was determined using a questionnaire on the occurrence of complaints (headache, nausea, stomach-ache, diarrhea, and other symptoms). Symptoms were graded on a 10-point scale with the grade 1 representing ‘Not present’, to 10 ‘Strongly present’. Subjects were asked to mark how they felt at that moment.

### Blood parameters

Blood samples were collected for analysis of Ghrelin, CCK, GLP-1, and PYY. The blood samples were mixed with EDTA to prevent clotting. The blood samples for CCK (5 ml) analysis were mixed with 2000 KIU Aprotinin (VWR International, Amsterdam, The Netherlands). Samples for GLP-1 (5 ml) were mixed with 40 µl of a dipeptidyl peptidase IV (DPP-IV) inhibitor (Linco Research Inc., St Charles, Missouri, USA) to prevent degradation. Plasma CCK levels were determined by a RIA (Euria-CCK, Eurodiagnostica AB, Malmö, Sweden). Plasma GLP-1, plasma total PYY levels, and plasma total Ghrelin levels were also determined by a RIA (Linco Research Inc., St Charles, Missouri, USA), all according to the manufacturers instructions.

### Statistical analysis

Data are presented as mean ± standard error of the mean (SEM), unless otherwise indicated. Statistical analysis was performed using SPSS 16.0 for Mac OS X software (SPSS Inc, Chicago, Illinois, USA). Mixed-model ANOVA was used to determine possible effects between the conditions. Differences in food intake between conditions and groups were calculated using the Students t-test. Correction for multiple comparisons was applied. All statistical tests were performed two-tailed and a p value of <0.05 was considered statistically significant.
